# Design and management considerations for control groups in hybrid effectiveness-implementation trials: Narrative review & case studies

**DOI:** 10.3389/frhs.2023.1059015

**Published:** 2023-03-10

**Authors:** Magdalena Jurczuk, Ranee Thakar, Fran E. Carroll, Lizzie Phillips, Jan van der Meulen, Ipek Gurol-Urganci, Nick Sevdalis

**Affiliations:** ^1^ Centre for Quality Improvement and Clinical Audit, Royal College of Obstetricians and Gynaecologists, London, United Kingdom; ^2^ Obstetrics & Gynaecology, Croydon University Hospitals NHS Trust, London, United Kingdom; ^3^ Maternity Services, University Hospital Plymouth NHS Trust, Plymouth, United Kingdom; ^4^ Department of Health Services Research and Policy, London School of Hygiene and Tropical Medicine, London, United Kingdom; ^5^ Centre for Implementation Science, Health Service and Population Research Department, King’s College London, London, United Kingdom

**Keywords:** study design, study management, hybrid trial designs, narrative review, comparative case study, implementation science, effectiveness-implementation

## Abstract

Hybrid effectiveness-implementation studies allow researchers to combine study of a clinical intervention's effectiveness with study of its implementation with the aim of accelerating the translation of evidence into practice. However, there currently exists limited guidance on how to design and manage such hybrid studies. This is particularly true for studies that include a comparison/control arm that, by design, receives less implementation support than the intervention arm. Lack of such guidance can present a challenge for researchers both in setting up but also in effectively managing participating sites in such trials. This paper uses a narrative review of the literature (Phase 1 of the research) and comparative case study of three studies (Phase 2 of the research) to identify common themes related to study design and management. Based on these, we comment and reflect on: (1) the balance that needs to be struck between fidelity to the study design and tailoring to emerging requests from participating sites as part of the research process, and (2) the modifications to the implementation strategies being evaluated. Hybrid trial teams should carefully consider the impact of design selection, trial management decisions, and any modifications to implementation processes and/or support on the delivery of a controlled evaluation. The rationale for these choices should be systematically reported to fill the gap in the literature.

## 1. Introduction

In the last decade, there has been a gradual shift in the field of implementation science towards combining the study of clinical effectiveness and implementation. This shift has the potential to reduce the translational gap between evidence and practice (1). To guide this change, a “hybrid” typology has been suggested that blends clinical effectiveness and implementation research, standardises the associated terminology, and distinguishes between the primary and secondary study aims positioned on the clinical effectiveness vs. implementation research continuum (2).

In brief, a type-1 design is most suitable for an intervention with a limited evidence base, where clinical effectiveness must be established. Implementation effectiveness is a secondary focus. On the other end of the spectrum is the type-3 design, more suitable for interventions that already have an evidence base for the clinical intervention's effectiveness, and therefore implementation is the main focus. In the middle is a type-2 design, in which clinical and implementation effectiveness outcomes share equal importance.

As hybrid studies are relatively new in the field of implementation research in health, there is a lack of guidance on how to optimally design and execute them. In addition to considering the appropriate hybrid type, researchers need to consider all “usual” decisions involved in study design (3). In practice, and also in our experience of designing, conducting and reviewing such studies, we would argue that it is a relatively new concept for a study's primary focus to be on *how* to implement rather than *what* to implement—which is the case for type-2 and type-3 hybrids. Where these types of studies have more than one study arm, these are distinguished not by the evidence-based practice (EBP) that is implemented, but rather by their “implementation mechanism”—in other words, the implementation support strategy (or bundle of strategies) prescribed as part of the study to roll out the EBP and be subject to evaluation.

Furthermore, it has recently been argued that implementation studies in general and hybrid studies in particular need to consider whether there is *contextual equipoise*, or “genuine uncertainty about whether the implementation strategies will effectively deliver the evidence-based practice in a new context”. Assessing contextual equipoise involves reflecting upon the evidence for a clinical intervention/programme to perform well at scale, and whether a control group is needed to evaluate a particular implementation mechanism (4). Although contextual equipoise evolved as a concept in the realm of global health implementation studies, it usefully triggers ethical questions that we believe are pertinent in thinking about the design of control groups in hybrid studies. A fundamental such question is whether there is sufficient evidence to suggest that a particular implementation support mechanism will be effective (or require only minor adaptation prior to application), or if a full-blown controlled evaluation is required to determine the optimal implementation mechanism. Where a controlled evaluation is required, a subsequent question is what type of control group is ethically warranted and practically scalable post-study–in other words, does one apply a traditional “no intervention” control, which means no implementation support whatsoever, or does one compare different implementation mechanisms, in the aspiration that the study will elucidate the one that is most optimal for post-study for scale-up, which might reasonably be the ultimate ambition of such a study. A further question might then become how to manage the delivery of the study, such that interventions that are being evaluated (i.e., the implementation mechanisms) are delivered in such a manner that upholds the prescribed comparisons and corresponding conclusions. Whilst this list of questions is indeed not exhaustive, it illustrates the need for some research and reflection around these issues to advance the design of hybrids. Although generic guides for the design of implementation studies have recently been published, these do not provide specific guidance on a suitable control group in hybrid trials, especially where implementation is a primary focus (5, 6).

The impetus for the present paper comes from an on-going hybrid type-3 study to improve maternity care and outcomes in Great Britain, referred to as “OASI2”. OASI2 has two study arms, one of which receives more implementation support than the other, in order to understand what is required for successful, scalable implementation of an evidence-based care bundle developed to reduce women's risk of childbirth injury (7). In the process of setting up and managing the trial, several decisions needed to be made related to the nature of the comparison arm; the nature of the implementation support strategies being evaluated; and how to support participating sites for optimal delivery of the study without deviating from the prescribed implementation mechanism and thereby compromising the study's evaluation objectives.

The work that we report here aimed to address some of the aforementioned questions in relation to the design and delivery of control groups in hybrid implementation evaluation. In Phase 1 of our work, we carried out a review of published hybrid type-3 designs that featured a comparison/control arm to understand how such studies are designed. In Phase 2, we then conducted a qualitative case study inquiry into different design and management choices and how case contexts have affected the implementation mechanism and study outcomes in three cases of hybrid evaluations: the OASI2, the “PA4E1”, and the “CATCH-UP” studies (see section 3.2, comparative case study overview for more detail) (7–9).

## 2. Methods

The research proceeded in two interlinked phases. In Phase 1, we carried out a review of the evidence base to identify how control groups have been operationalized in hybrid-3 studies and develop a de facto typology. Based on what we found, in Phase 2 we selected a number of exemplar case studies, which we examined in detail to enable a reflection on the nature and application within research contexts of different types of control groups.

### 2.1. Phase 1: narrative review

A narrative review of hybrid trials was conducted to identify hybrid type-3 trials involving comparison/control and intervention groups with well-defined implementation support strategies. On 8-February 2022, PubMed was searched for publications with the following words in the title and/or abstract: “hybrid”, “effectiveness”, “implementation”, limiting the search to studies published in the English language. The search was intentionally broad and did not specify type-3 trials as many studies are reported as hybrid without stipulating the type. Titles and abstracts of the resulting publications were then reviewed for relevance, excluding non-hybrid studies. Hybrid studies without a specified type were assessed by the lead author (MJ) to deduce the type where possible. Publications of hybrid type-3 trials were then reviewed to identify different design structures. Information on the area of study, the intervention/programme being implemented, and implementation support strategies across the different study arms were extracted.

### 2.2. Phase 2: comparative case study analysis

For each design structure identified in the narrative review, one case study was selected for the comparative case study analysis, to be featured alongside the OASI2 study, which is led by our research group. Various approaches to case study methodology have been applied in the realm of implementation science (10). Our analysis is grounded in the theories of a realist evaluation, where the focus is on how context case-specific contexts influence the implementation mechanism and eventual study outcomes (10).

All published literature on the additional cases were reviewed and follow-up questions specific to the aims of this paper were developed for a semi-structured interview guide (i.e., how was the comparison/control arm managed in practice? Did the study team experience any challenges related to managing the comparison/control arm? Was scalability of the implementation strategies taken into account during the design? Were any changes made to implementation tools?). Lead authors of the selected case studies were invited to participate in a brief semi-structured phone interview with the lead author (MJ). The full set of follow-up questions can be found in Supplementary Data Sheet 1.

Common themes related to implementation support strategies, study arm management, and support offered to participating sites were identified from the publications and follow-up calls.

## 3. Results

### 3.1. Phase 1: narrative review

The search yielded 670 publications, excluding the OASI2 study protocol. Titles and abstracts were reviewed for relevance and categorised by type of hybrid trial. 224 publications were initially excluded as not relevant (not reporting on a hybrid implementation effectiveness trial). 446 articles were about hybrid trials, and 101 of these did not specify the type. The lead author (MJ) deduced the type of 79 of these 101 unclassified publications based on the description of the study design and outcomes. Of the 424 publications that were classifiable to a defined hybrid type, 183 were type-1, 156 were type-2, and 85 were type-3.

The methods of the 85 publications on hybrid type-3 trials were subsequently assessed to determine if a comparison/control arm was featured, and 32 were excluded on this basis. Of the resulting 53 in-scope publications, eight were excluded as they reported on a trial already represented by another publication.

**Figure 1:**
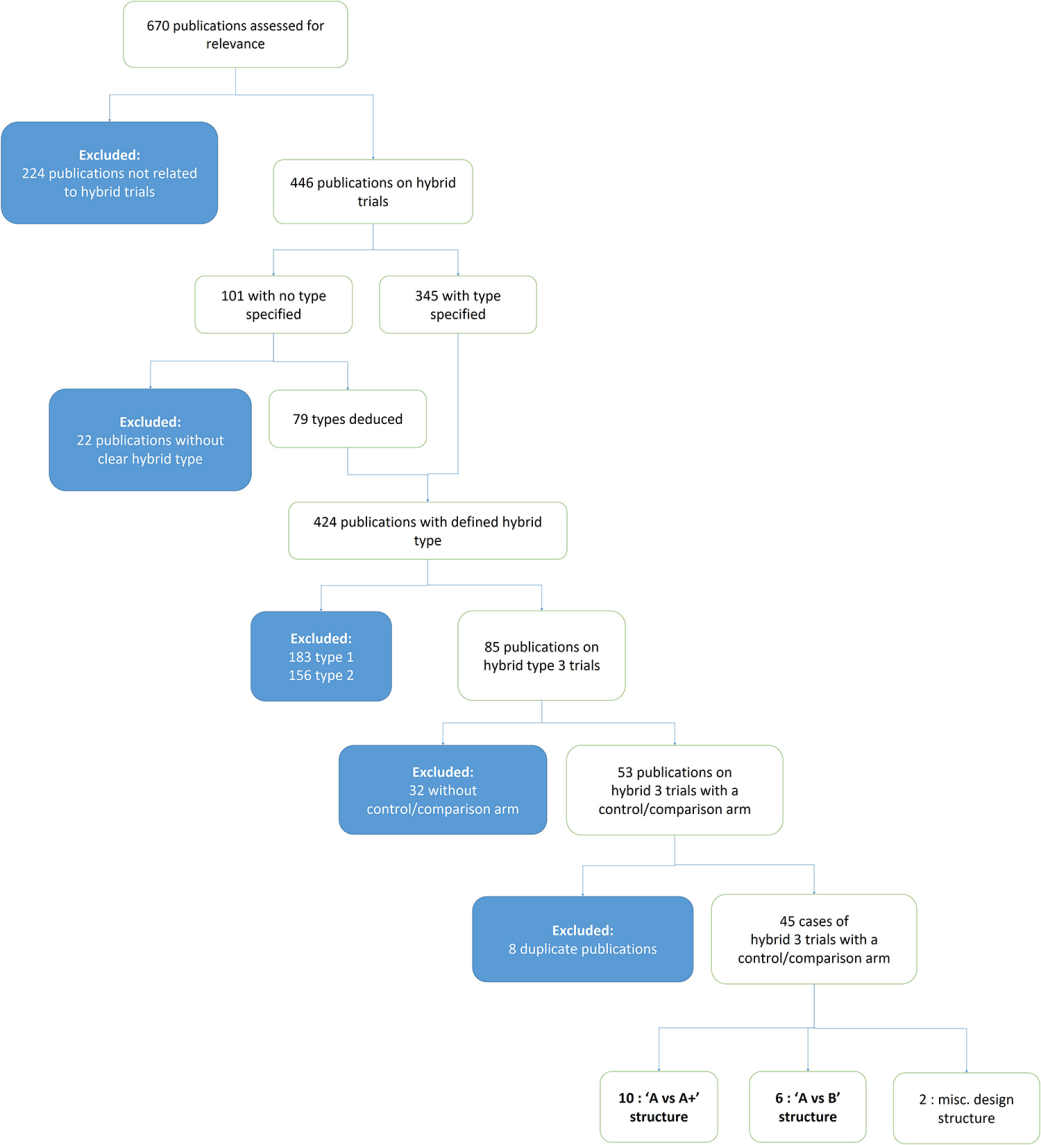
Narrative review process.

The resulting 45 publications on distinct hybrid type-3 trials formed the corpus of the review. These reports were reviewed to identify the most common design structures. Figure 1 depicts how publications were assessed and selected for inclusion in the review.

17 had an “A vs. A+” structure (9, 11–26), 26 had an “A vs. B” structure (8, 27–51) and 2 had unique structures that did not fall into the two defined categories (52, 53).

Three categories of study design structures were identified across the 45 type-3 cases: 17 had an “A vs. A+” structure, 26 had an “A vs. B” structure and 2 had unique structures that did not fall into the two defined categories. In “A vs. A+”, both study arms share a baseline mechanism of implementation (“A”) and one arm receives additional support (“A+”). In an “A vs. B” structure, one mechanism of implementation (“A”- i.e., an implementation strategy or combination of strategies) is compared to a different mechanism or no mechanism offered to the study sites at all (“B”) without overlap between the two. This includes the traditional intervention vs. “usual practice” controlled designs as well as stepped wedge trials. See Supplementary Table 1 for a detailed presentation of the 45 type-3 publications from which the case studies were selected.

The motivation for this narrative review was to compare studies with similar design to OASI2,which compares supported vs. unsupported facilitation of EBP implementation in Great Britain. Studies that were not comparable were not considered in the selection for comparative case study: these included studies taking place in low and middle income countries (4 papers), studies that did not involve facilitation as an implementation strategy (13 papers), studies with a unique design structure (2 papers), and studies with a stepped wedge design (10 papers). The rationale for excluding stepped wedge trials in particular was that one of the predicaments raised by the OASI2 team during study design was whether the unsupported (control arm) sites would continue their participation in the study without the promise of eventually receiving full implementation support (as would happen by design in a stepped wedge trial) – this would be a fundamentally different scenario.

One exemplar case study for each was selected to represent the ten remaining “A vs. A+” and six remaining “A vs. B” structures, respectively. The case exemplar selected for the “A vs. A+” structure was CATCH-UP, a trial implementing a health insurance enrolment tracking tool in community health centres (CHCs) in the United States (9). The case exemplar selected for the “A vs. B” structure was the scale-up of the “Physical Activity for Everyone” (PA4E1) programme, which promotes adolescent physical activity.

Table 1 gives an overview of these two cases alongside the OASI2 study.

**Table 1:** Summary of cases representing hybrid type-3 trials with a comparison/control arm.

Category of hybrid type-3 trial	Case	Clinical intervention	Site of implementation	Comparison/control arm	Intervention arm
A vs. B	P4AE1^a^	Physical activity programme	Secondary schools in New South Wales, Australia	Received no additional support/materials	Internal facilitator trained to support each school throughout the study to implement a bundle of implementation strategies for programme roll out
A vs. A+	CATCHUP^b^	Insurance re-enrolment tool	Community Health Clinics in the US	Received educational materials only	Received educational materials + an external practice facilitator offering on-site, face-to-face trainings and support.
A vs. A+	OASI2^c^	Care Bundle to prevent childbirth injury	20 NHS maternity units in Great Britain	Received implementation toolkit and required to nominate two internal facilitators prior to study start	Received implementation toolkit, required to nominate two internal facilitators prior to study start, external experienced facilitator from within the same region to offer continuous support

^a^ Physical Activity 4 Everyone Programme (scale-up study).

^b^ Community-based Health Information Technology (HIT) Tools for Cancer Screening and Health Insurance Promotion (implementation study).

^c^ Obstetric Anal Sphincter Injury 2 (OASI Care Bundle scale-up study).

### 3.2. Phase 2: comparative case study overview

In this section, we describe the context of each study, including the study design and how this affected the intended implementation of the EBP and the study outcomes.

#### 3.2.1. OASI2 study

OASI2 is a cluster randomised controlled trial (C-RCT) that seeks to understand what is required for scalable implementation of the OASI Care Bundle (the EBP), a set of 4 evidence-based practices to reduce women's risk, and improve detection of severe tearing during childbirth (54). OASI2 has two randomised study arms, each comprised of 10 National Health Service (NHS) maternity units without prior experience of implementing the OASI Care Bundle. A third, parallel arm of 10 maternity units that had previously implemented the care bundle as part of the preceding study (OASI1) is engaged to offer external facilitation to one of the randomised arms as well as to allow further evaluation of the care bundle's sustainability over time.

An implementation toolkit was developed to guide all participating sites with EBP roll out, incorporating lessons learned from the preceding OASI1 study's process evaluation. The toolkit includes a clinical manual, an implementation guidebook for local facilitators, updated awareness campaign materials, and an eLearning package. All participating sites were also required to select two local facilitators, or “leads” to be responsible for local implementation.

The intervention arm is called the “peer support” arm. Leads from peer-supported units receive external experienced facilitation from OASI1 sites in the parallel arm. The comparison arm is the “lean implementation” arm. Leads from the lean implementation units do not receive any external support. Units in both the peer support and lean implementation arms receive the implementation toolkit.

In effect, the key difference between the two C-RCT arms is that the peer support arm receives continuous external support, while the lean implementation arm does not. The participating sites’ individual contexts (i.e., preparedness of selected leads to facilitate local implementation, senior/institutional support, and staff's pre-existing acceptance of the EBP) had an impact on how well the intended implementation mechanism was carried out. Deviations from the intended implementation mechanism include peer supported sites not engaging with their external facilitators (which can be construed as reduced fidelity of receipt of the intended implementation support intervention) and lean sites reaching out to the study team for additional support (likewise, due to the potential for increased implementation support beyond what was intended as per study design; and also potential for contamination, if the study team inadvertently functioned as “peer support” to the lean/control sites).

#### 3.2.2. CATCH up study

The Community-based Health Insurance Technology (HIT) Tools for Cancer Screening and Health Insurance Promotion (CATCH-UP) intervention seeks to increase cancer screening and prevention care in uninsured patients in community healthcare settings in the United States. The CATCH-UP study included 23 community health clinics that implemented the HIT tool (the EBP).

The 23 participating clinics were randomised to two study arms. Clinics in the intervention arm received educational materials (electronic manual with instructions for tool use), beta testing, and a practice facilitator to explain the insurance support HIT tools, prepare clinic staff for using the tools, and assist clinics in revising workflows. The practice facilitator was available to the clinic staff and continued to engage actively during the implementation phase, including on-site trainings and support. Clinics in the comparison arm received educational materials only.

As there was no preceding study to establish the tool's effectiveness, the 23 clinics were matched with comparison sites to test the effectiveness of the tool—effectiveness was established based on outcomes in the 23 clinics in the intervention and comparison arms compared to the 23 clinics in the externally matched control group.

The implementation component of the trial compared the two levels of implementation support in the two arms, evaluating acceptance and use of the tools, as well as patient, provider, and system level factors associated with successful implementation of the tools (55, 56).

This study's context necessitated a modification to the CATCH-UP trial's insurance support tool (the EBP itself): as one of the main benefits for clinics to implement and use the tool was that it provided support with completing a complex report required by an external funding agency. Before the trial ended, the funding agency simplified its reporting requirements, decreasing the tool's value. The CATCH-UP trial team therefore had to adapt to this change by modifying the tool itself. The context had a major impact on the composition of the EBP and, by default, on how it was implemented.

#### 3.2.3. PA4E1 scale-up study

“Physical Activity 4 Everyone” (PA4E1) is a multi-component, secondary school-based intervention promoting adolescents' physical activity in New South Wales, Australia. PA4E1 was first evaluated in a preceding study where it was found to be effective in slowing the decline in physical activity of adolescents at disadvantaged schools when compared to the control group, which carried on with usual practice (57).

In the PA4E1 scale-up study, the physical activity programme (the intervention) remained largely the same as in the preceding efficacy study, except that the six implementation strategies were slightly adapted to support delivery at scale and to synchronise with existing systems (57–60). One strategy—internal facilitation—was added as a seventh strategy as this was identified as a cost effective model for scalable delivery (61).

A total of 76 schools were randomised into the intervention or control arms. Schools in the intervention arm received the scalable implementation support (the seven strategies) to implement PA4E1. Schools in the control arm were introduced to PA4E1 at the beginning of the study *via* a brief presentation. They did not receive additional implementation support apart from usual care, which was reactive support based on explicit requests from the schools.

An electronic portal gave participating sites access to the implementation materials. Schools in the control arm had restricted access and could only see an overview of the school physical activity programme, while schools in the intervention arm could access all of the content, including a resource manual and online training (8).

In this study, contextual factors were not reported to have impacted on the EBP itself nor on how it was implemented. Both the EBP and the implementation mechanism were rolled out as originally intended by the study team.

#### 3.2.4. Reflection on design challenges and responses

All three hybrid case exemplars have experienced a tension on the spectrum between fidelity to the original study design and reactively tailoring to the unique needs of participating sites that surface during the study conduct.

In OASI2, the tension manifested in two ways: in choosing how to respond to requests from participating sites that were contradictory to the prescribed implementation mechanism, and in deciding whether to make changes to the implementation toolkit based on participants' feedback before trial's end. Since the start of the trial local leads from the lean implementation arm (comparison arm) have reached out to the study team for additional guidance on how to implement the care bundle, a request in direct opposition with the “lean” nature of the arm. Similarly, there have been instances where leads in the peer support arm were not receiving external facilitation as planned. Additionally, since awareness of the OASI Care Bundle intervention had spread throughout NHS maternity units after the OASI1 study; non-participating sites began reaching out with requests to receive the toolkit before the OASI2 study launched. There was a surge of external requests around the summer of 2021, when the NHS launched an initiative to roll out new pelvic health clinics (62).

The study team convened to discuss how to address each scenario, weighing research design and evaluation considerations against “meeting the moment” and catering to the requests of both participating and non-participating sites.

Concerning the lean implementation (comparison) arm, we opted to signpost back to the toolkit. With regards to enforcing peer support in the intervention arm, we clarified expectations with the external facilitators and reminded them of their role as often as possible. In addition, we reached out to all peer supported units to investigate if they felt satisfied with the support offered to them since the start of the study. Finally, in response to sharing the implementation materials externally, we opted to wait until all participating units had the toolkit before making it publicly available *via* an online request form.

After the study had launched and the toolkit was disseminated, toolkit users reached out with suggested modifications to the resources to improve their usability. The team opted to make the suggested changes in the first half of the study, recognizing that the alternative option of waiting until the end of the study might negatively impact the EBP's uptake. The toolkit materials will undergo further modification to address all other feedback received at the outset of the study, with a goal to create an updated toolkit that is publicly available after the trial is over.

In the CATCH-UP trial, staff from clinics in the comparison arm (meant to receive educational materials only) reached out to the study team asking for additional guidance. Similarly to the OASI2 team's response to its comparison arm, the CATCH-UP team opted to address these requests by organising a meeting with the clinic staff and answering their questions by signposting back to the educational materials originally provided. The CATCH-UP study team recognised during the management of the study that there was a choice to be made between not engaging (per the study design), or responding to these comparison arm clinics, which, as evidenced by their request for help, were motivated to succeed. In this instance, the team opted to engage with the site, though without offering more support than what was originally intended.

In the PA4E1 study, the research team not only recognised but foresaw the tension between fidelity and tailoring. In anticipation of some participating schools in the intervention group failing to meet programme milestones, they created an external facilitator role responsible for intervening with the underperforming schools' executive leadership to identify the key barriers and enablers. In this case, reactive tailoring was built into the implementation support as originally intended.

## 4. Discussion and recommendations

Our review illustrates that despite the lack of clear guidance on how to design and manage hybrid trials, particularly related to comparison/control arms and managing pragmatic challenges to the study design, such studies are increasingly prevalent: 86% (366 of 424) publications on hybrid trials that we identified were published in the last five years (since 2018).

The narrative review identified two dominant patterns in the design of study arms. The A vs. B design structure seeks to identify the “best overall” implementation strategy or set of strategies when compared to another set of strategies or a “true” control, while the A vs. A + design structure aims to evaluate the value of the additional support over and above “basic”, “no-frills” implementation support approaches. We recommend that to guide selection of design structure in the early stages of trial development, researchers should internally assess contextual equipoise and beneficence in terms of ensuring that no study participants/sites are denied strategies with sufficient evidence of effectiveness (4). We realise that a judgement of sufficiency will always risk being subjective and that the gold standard to determine strength of evidence to support use of an implementation strategy or bundle of strategies will be a controlled study. However, this ought to be balanced against the need to implement well-evidenced EBPs speedily and sustainably. Hybrid implementation studies require time and resource, so to avoid the risk of ever extending the time lag between evidence and practice decision-making regarding what implementation mechanisms may require trialing and how we argue here that pragmatism and true contextual equipoise ought to be considered. Researchers should also include the rationale for their decision-making and ultimate selection of comparators/controls in study protocols to support the informed design of future trials.

Our case study analysis suggests that the OASI2, CATCH-UP, and PA4E1 study teams all recognised that their trial management required striking the right balance between fidelity to the study design and catering to the needs of participating sites. At its extreme, choosing fidelity to study design means withholding support or even contact with some sites. The foreseeable consequences of this could include sites losing interest in the trial, failing to collect data required for evaluation or even withdrawal from the study. Ethical considerations of withholding support should also be considered. The other extreme is catering to every emergent request from a participating site, regardless of study arm allocation. The consequence of this is a trial that is unable to make any claims about how to effectively implement an intervention sustainably and at scale. We argue that neither extreme is beneficial and therefore finding an optimal balance on this spectrum is vital. Researchers working on similar hybrid trials should reflect on the crossroad decisions made throughout the trial and include commentary on trial management in papers reporting study results.

All three case exemplars we report also demonstrate that modification—either of the intervention being implemented or the implementation strategies being evaluated—can be necessary and unavoidable. Although modification(s) may lead to improved implementation outcomes, it can also make evaluation challenging. To mitigate this, systematically recording these modifications is essential. The Framework for Reporting Adaptations and Modifications-Enhanced (FRAME) was developed to support the reporting of modifications made to interventions (63), and more recently, FRAME has been adapted to support reporting modifications to implementation strategies (64). All implementation trials, but especially hybrid trials, should report modifications using FRAME in results papers.

## 5. Limitations

The narrative review has a limited scope as the intention was to identify cases comparable to the OASI2 study. Although stepped wedge designs were excluded, they may offer valuable insight regarding optimal design and trial management decisions in hybrid trials. The 32 in-scope cases (refer to Supplementary Table 1) were reviewed to determine the design structure and only two study teams (CATCH UP and PA4E1) were contacted for follow-up discussion.

## 6. Conclusion

In the interest of contributing to the development of guidance on design and management of hybrid trials, systematic reporting of rationale for design selection, crossroad decisions during the trial, and any modifications made to the intervention or implementation strategies should become routine.
