# Supplementary material for: Design and management considerations for control groups in hybrid effectiveness-implementation trials: Narrative review & case studies
Source: Front Health Serv. 2023 Mar 10;3:1059015. doi: 10.3389/frhs.2023.1059015 (PMC10012616; doi:10.3389/frhs.2023.1059015)
Supplement: Supplementary file 2 [file Datasheet1.pdf]

Questions about the CATCH UP study

1. The main difference between your two study arms is that one of the arms includes a practice facilitator who is available to the clinic staff and engages actively throughout the implementation phase, including on-site, face-to-face trainings and support. The 'peer support' arm in OASI2 is very similar in that way. We were wondering whether the practice facilitator is part of clinic staff or part of the study team (or otherwise)? Secondly, when designing the study, was scalability of this type of facilitation taken into account? In other words, did your team know whether facilitation is something that could be offered by your collaborators at OCHIN if it turned out that practice facilitation was needed to ensure successful implementation of the tool?

2. Can you describe how you managed the materials-only/no-facilitation arm of your study? What type of contact did you have with them throughout the study? Was communication strictly limited to data collection matters? Your team visited sites across both study arms. Did you experience any challenges with avoiding inadvertent facilitation during the visits? Did you also visit the sites that were in the matched control group?

3. Your study tested both initial effectiveness of the tool and implementation methods. Did they experience any resistance to implementation due to lack of effectiveness evidence? If you were to do it again, would you do it the same way, or would you consider testing effectiveness separately before focusing on implementation? Why?

4. Refinement of the tool and educational materials: I understand that in the beginning of the study, the tool itself was getting refined to fit better within existing workflows of the participating sites. Assuming all sites work differently, did you tailor the tool to each site, or maintain a standardised version of the tool that was suitable to all? Did you refine the tool again at the outset of the study, in accordance with new findings? Were the educational materials refined as well? Now that the study is over, can other interested sites access the tool and the corresponding materials?

Questions about the PA4E1 scale up study

1. Can you describe how you managed the control arm of your study? What type of contact did you have with them throughout the study? Was communication strictly limited to data collection matters? Were there any challenges to doing this? Was it difficult to abstain from offering implementation advice? Did schools in the control group already know about PA4E1 from the preceding study?

2. My understanding is that your study compared the number of schools that implemented at least 4 of the 7 practices of PA4E1 program in the intervention group vs the control group. Assuming a best case scenario involving a really high performing school in the control arm that

is very motivated to increase kids' physical activity, how would that school implement PA4E1? Did they have access to any information on what PA4E1 is?

3. You noted in your protocol that the program resources would be made available to the control group upon study completion. Can you clarify which program resources those are? Did your team consider making just the resources (and no other implementation support) available to the control group during the study?

4. Since the completion of the scale up trial, were the overarching implementation support strategies further refined based on new findings? Are any resources (the ones used in the scale up study or any updated versions) available more widely to other schools interested in implementing the program?
